# The impact of the coronavirus disease and Tele-Heart Failure Clinic on cardiovascular mortality and heart failure hospitalization in ambulatory patients with heart failure

**DOI:** 10.1371/journal.pone.0249043

**Published:** 2021-03-23

**Authors:** Sarinya Puwanant, Supanee Sinphurmsukskul, Laddawan Krailak, Pavinee Nakaviroj, Noppawan Boonbumrong, Sarawut Siwamogsatham, Krailerk Chettakulanurak, Aekarach Ariyachaipanich, Smonporn Boonyaratavej

**Affiliations:** 1 Division of Cardiology, Department of Medicine, Faculty of Medicine, Chulalongkorn University, Bangkok, Thailand; 2 Cardiac Center, King Chulalongkorn Memorial Hospital, Thai Red Cross Society, Bangkok, Thailand; Maastricht University Medical Center, NETHERLANDS

## Abstract

**Background:**

We sought to investigate the impact of the COVID-19 pandemic and the Tele-HF Clinic (Tele-HFC) program on cardiovascular death, heart failure (HF) rehospitalization, and heart transplantation rates in a cohort of ambulatory HF patients during and after the peak of the pandemic.

**Methods:**

Using the HF clinic database, we compared data of patients with HF before, during, and after the peak of the pandemic (January 1 to March 17 [pre-COVID], March 17 to May 31 [peak-COVID], and June 1 to October 1 [post-COVID]). During peak-COVID, all patients were managed by Tele-HFC or hospitalization. After June 1, patients chose either a face-to-face clinic visit or a continuous tele-clinic visit.

**Results:**

Cardiovascular death and medical titration rates were similar in peak-COVID compared with all other periods. HF readmission rates were significantly lower in peak-COVID (8.7% vs. 2.5%, p<0.001) and slightly increased (3.5%) post-COVID. Heart transplant rates were substantially increased in post-COVID (4.5% vs. peak-COVID [0%], p = 0.002). After June 1, 38% of patients continued with the Tele-HFC program. Patients managed by the Tele-HFC program for <6 months were less likely to have HF with reduced ejection fraction (73% vs. 54%, p = 0.005) and stage-D HF (33% vs. 14%, p = 0.001), and more likely to achieve the target neurohormonal blockade dose (p<0.01), compared with the ≥6-month Tele-HFC group.

**Conclusions:**

HF rehospitalization and transplant rates significantly declined during the pandemic in ambulatory care of HF. However, reduction in these rates did not affect subsequent 5-month hospitalization and cardiovascular mortality in the setting of Tele-HFC program and continuum of advanced HF therapies.

## Introduction

The coronavirus disease (COVID-19) is an emerging contagious disease affecting patients, health care providers, and the health care system worldwide [1, 2]. Previous studies [3, 4] have shown that the incidence of hospitalization for heart failure (HF) in acute HF patients significantly declined during the peak of the COVID-19 pandemic. However, little is known about the adverse outcomes after the pandemic lockdown. Furthermore, there has been a paucity of data about the impact of COVID-19 on ambulatory patients with chronic HF. We sought to investigate cardiovascular (CV) death, HF hospitalization, and heart transplantation rates in ambulatory patients with HF before, during, and after the peak of the pandemic and to examine the effect of tele/virtual visits on those outcomes.

## Materials and methods

### Study design and population

The study protocol was approved by the Institutional Review Board of the Faculty of Medicine, Chulalongkorn University. Using the HF clinic database, data of 234 patients with HF who were actively followed up in the advanced HF clinic in King Chulalongkorn Memorial Hospital between March 17 and May 31, 2020 were analyzed. We excluded patients with a left ventricular assist device (n = 4) and patients who had been managed in the HF clinic for less than 3 months (n = 29). A total of 201 patients were included in the analysis. We compared data of patients with HF before, during, and after the peak of the pandemic (January 1 to March 17 [pre-COVID], March 17 to May 31 [peak-COVID], and June 1 to October 1 [post-COVID]). To exclude the potential confounding effect of the seasonal trends in HF hospitalization, case referral, and transplantation, we compared data of these 3 study periods with the corresponding periods in 2019. Heart failure with reduced ejection fraction (HF-rEF) was defined as a clinical diagnosis of heart failure with a left ventricular ejection fraction (LVEF) ≤ of 40% [5]. Heart failure with improved ejection fraction (HF-iEF) was defined as a clinical diagnosis of heart failure with a left ventricular ejection fraction (EF) ≤ 40% at baseline, combined with a ≥10% absolute improvement in LVEF and a second LVEF measurement > 40% [6]. Stage D heart failure was defined as advanced heart failure with clinical events and findings, including frequent heart failure hospitalization, progressive deterioration of renal function, intolerance to neurohormonal blockades, frequent hypotension, persistent dyspnea, requirement of a high dose of diuretic to maintain fluid status, hyponatremia or progressive decline in serum sodium, high natriuretic peptide, frequent ICD shocks, or peak oxygen consumption < 12–14 ml/kg/min, as previously described [5].

### Tele-Heart Failure Clinic program during the coronavirus disease pandemic

Between March 17 and May 31, 2020, all patients in the HF clinic were managed by the Tele-HF Clinic (Tele-HFC) program. The Tele-HFC program offered therapeutic intervention and regular virtual visits, including audio/telephone and video conferencing, based on the patients’ preferences. Patients who received therapeutic interventions were followed up tri-weekly or more frequently depending upon clinical circumstances, while patients stable on unchanged HF medication were followed up on an every-3-month-basis. Patients falling between the two groups were followed up bi-monthly or more frequently depending upon the clinical status. Patients were required to electronically submit their daily symptom checklists, body weight, blood pressure, heart rate, and blood test results, if indicated, to the HF nurses. Of the study patients, 98% had weight scales and 66% had a home blood pressure monitor. The management decision was made by a team-focused approach consisting of HF and transplant cardiologists, HF specialist nurses, and HF pharmacists. After June 1, patients could choose either a face-to-face clinic visit or a continuous tele-clinic visit.

### Outcome measures

Outcomes included CV death, heart transplantation, HF hospitalization, or medical uptitration rates of neurohormonal blockades. Outcomes were determined by reviewing medical records, death certificates, and current obituaries, and making telephone calls. All patients were followed up until death or October 1, 2020, when the patients were censored. Survival and clinical outcome data were available for all study patients.

### Statistical analysis

Continuous data were analyzed using the paired t-test or Wilcoxon signed-rank test depending on their distribution. Categorical data were analyzed using the chi-squared test or Fisher’s exact test, where appropriate. Statistical significance was defined as a p-value of <0.05.

## Results

### Baseline characteristics

Table 1 shows the baseline characteristics of study patients with HF compared with the cohort in the corresponding periods in 2019. Among 201 patients during the pandemic, 133 (66%) had HF with reduced ejection fraction (HF-rEF), 22 (11%) had HF with improved ejection fraction, and 45 (22%) had stage-D HF. Patients in the cohort of 2019 had similar mean age, mean left ventricular ejection fraction (LVEF), the proportion of stage-D HF, and proportion of New York Heart Association (NYHA) functional class III to IV.

**Table 1:** Baseline clinical characteristics.

	Year 2020	Year 2019	p-Value
**No. of patients** **(All patients)**	**201**	**269**	
Age (years)	56 ± 16	54 ± 17	0.195
Male, no. (%)	135 (67%)	195 (72%)	0.212
Left ventricular ejection fraction (%)	37 ± 18	39 ± 23	0.437
Ischemic, no. (%)	73 (36%)	96 (36%)	0.887
NYHA III-IV, no (%)	40 (20%)	49 (18%)	0.645
Stage-D HF, no (%)	45 (22%)	73 (27%)	0.240
HF with reduced EF/ HF with improved EF, no. (%)	133/22 (66/11%)	183/15 (68/6%)	0.671
Atrial fibrillation, no. (%)	57 (29%)	63 (24%)	0.243
Furosemide >160 mg/day, no (%)	27 (13%)	46 (17%)	0.280
Thiazide/tolvaptan, no (%)	14 (7%)	16 (6%)	0.655
BUN (mg/dL)	23 ± 12	26 ±17	0.650
Creatinine (mg/dL)	1.45 ± 1.57	1.56 ± 2.08	0.453
NT-proBNP (pg/mL)	10 299 ±8092	7382 ± 8310	0.166
**No. of patients** **(Patients with HF-rEF or HF-iEF)**	**155**	**198**	
ACEI/ARB/ARNI, no. (%*)	138 (89%)	170 (86%)	0.375
Beta-blockers, no. (%*)	145 (94%)	180 (91%)	0.363
MRA, no. (%*)	95 (61%)	116 (59%)	0.607
CIED, no. (%*)	65 (42%)	63 (32%)	0.040

ACE-I indicates angiotensin-converting enzyme inhibitor; ARB, angiotensin receptor blockade; ARNI, angiotensin receptor neprilysin inhibitor; BNP, B-type natriuretic peptide; BUN, blood urea nitrogen; CIED, cardiac implantable electronic device; EF, ejection fraction; HF, heart failure; MRA, mineralocorticoid receptor antagonist; and NYHA, New York Heart Association functional class.

* Percentage calculated from the number of patients with HF with reduced EF or HF with improved EF (n = 155 in 2020 and n = 198 in 2019).

### Pre-COVID-19 versus peak-COVID-19 versus post-COVID-19

**Fig 1:**
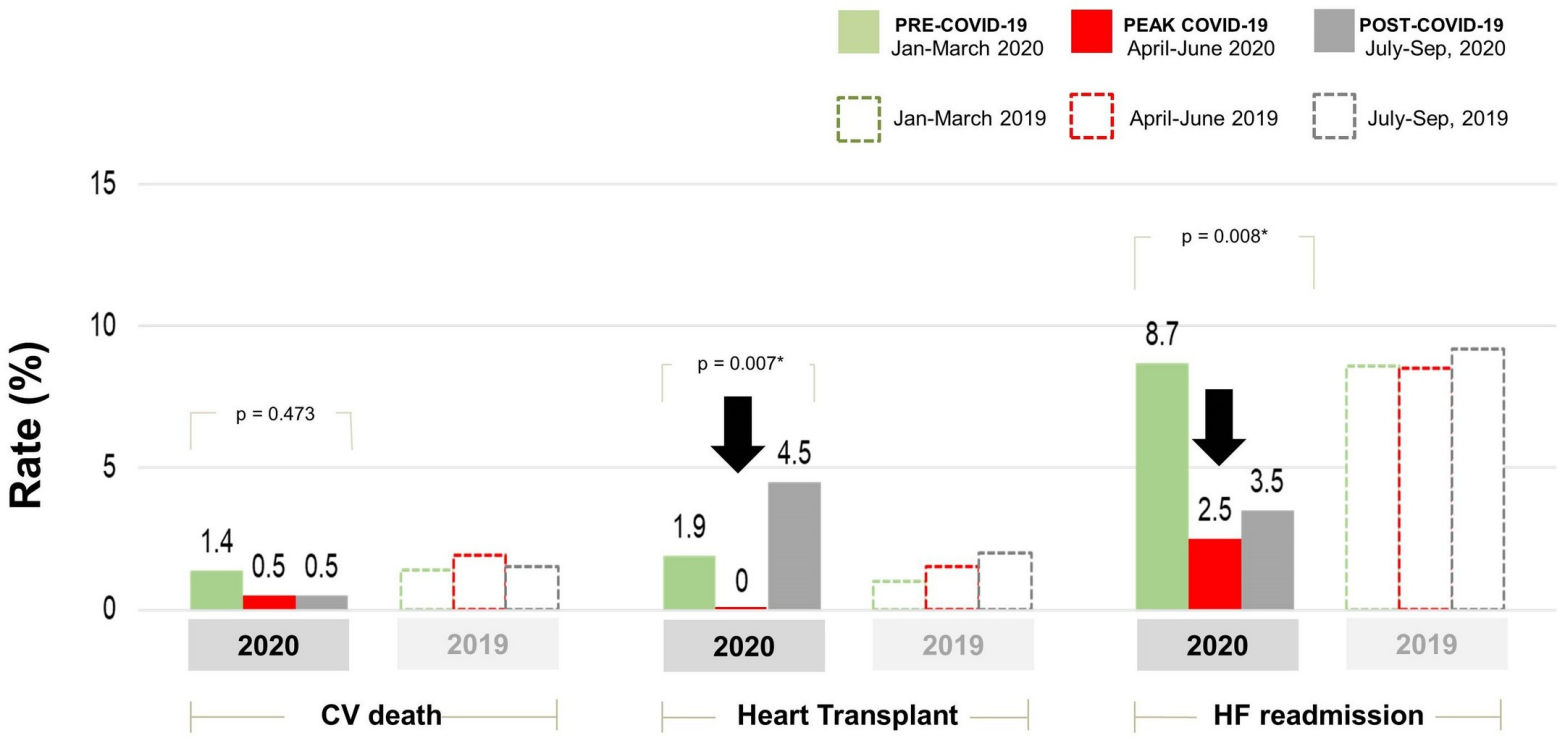
Rates of cardiovascular mortality, heart failure hospitalization, and heart transplantation during pre-COVID-19, peak COVID-19, and post-COVID-19 periods of 2020 and corresponding periods in 2019.

**Fig 2:**
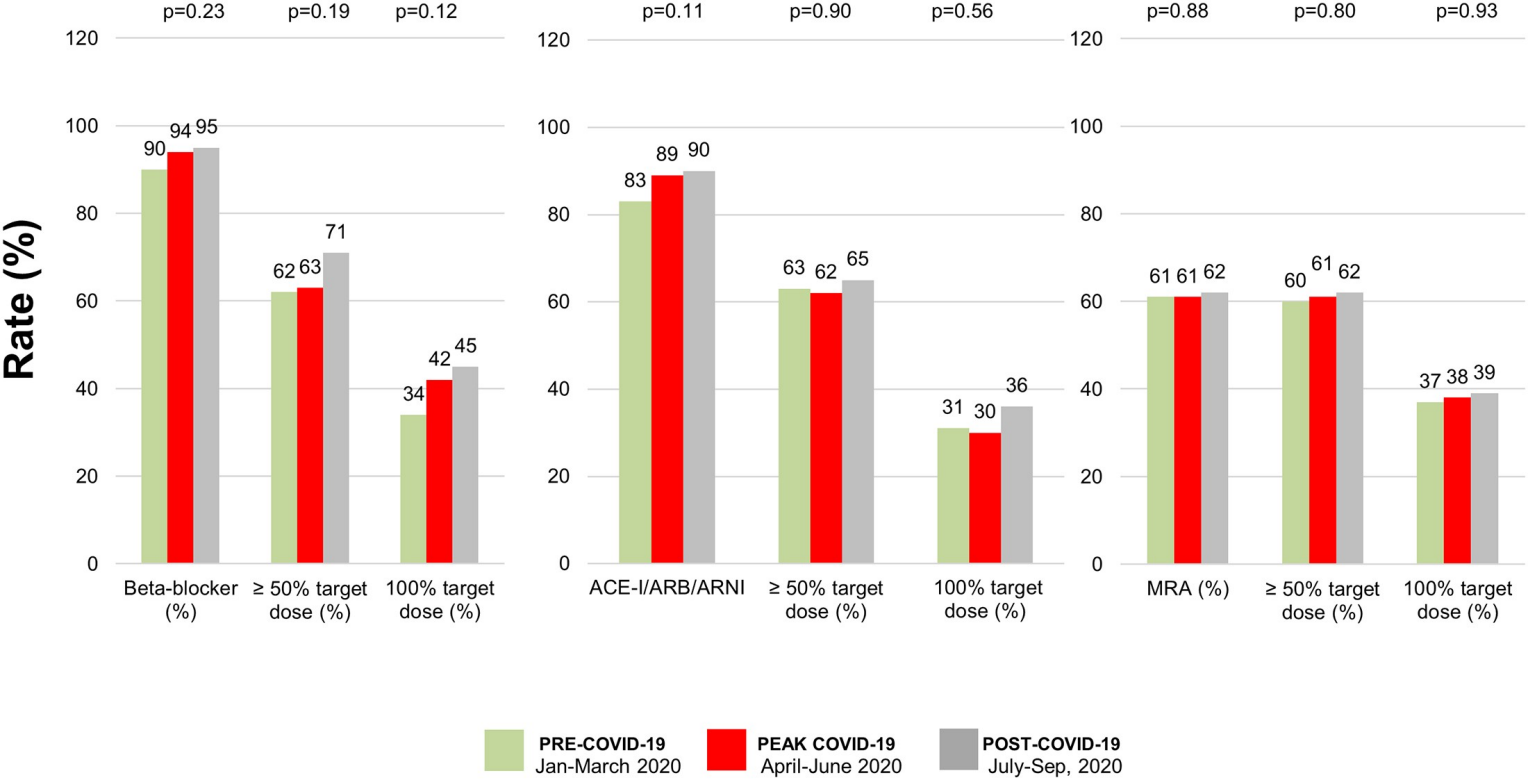
Rates of medical titration during pre-COVID-19, peak COVID-19, and post-COVID-19 periods of 2020.

Fig 1 illustrates cardiovascular death, HF readmission, and transplantation rates across the 3 study periods (January 1 to March 17 or pre-COVID [n = 208], March 17 to May 31 or peak-COVID [n = 201], and June 1 to October 1 or post-COVID [n = 200]). CV death rates during the peak of the pandemic were similar in comparison with all other periods (1.4% (3/208) in the pre-COVID period, 0.5% (1/201) at the peak of the COVID-19 pandemic, and 0.5% (1/200) post-COVID, p = 0.473). The heart transplant program was suspended during March-May 2020. Therefore, heart transplant rates declined from 1.9% (4/208) in the pre-COVID period to zero at the peak of the pandemic and substantially increased in the post-COVID-19 pandemic period (0% during the peak of the pandemic versus 4.5% (9/200) after the peak of the COVID-19 period, p = 0007). HF readmission rates were significantly lower in the peak-COVID period (8.7% (18/208) versus 2.5% (5/201), p<0.001) and slightly increased (3.5% [7/200]) in the post-COVID period. One patient in our cohort had a diagnosis of COVID-19 but was asymptomatic with an uneventful outcome. Rates of neurohormonal blockade uptitration during the peak of the pandemic were not different from all other periods (Fig 2).

### Year 2019 versus year 2020

Fig 1 also illustrates HF outcomes in the corresponding 3 time periods (January 1 to March 17, March 17 to May 31, and June 1 to October 1) in 2019. We observed that HF readmissions significantly decreased during the peak of the COVID-19 pandemic and remained lower than those in the 2019 cohort after the peak of the pandemic. However, CV deaths were similar in both cohorts. The distribution of heart transplants in 2019 was not affected by seasonal trends.

### Short-term versus long-term Tele-Heart Failure Clinic program

After June 1, 75 patients (38%) preferred to continue with the Tele-HFC program. Table 2 shows patient characteristics in the short-duration (<6 months) and longer-duration (≥6 months) Tele-HFC groups. Median time on the Tele-HFC program in both groups was 136 (4–160) days and 183 (180–195) days. Patients who were managed by at least 6 months of Tele-HFC were more likely to have stable chronic HF, less likely to have stage-D defined HF (14% versus 33%, p = 0.001) and HF-rEF (59% versus 76%, p = 0.01), less likely to be in advanced NYHA class (11% versus 24%, p = 0.017), less likely to receive a large dose of diuretics (13% versus 23%, p = 0.117), less likely to require additional diuretic or aquaretic therapy (1% versus 15%, p = 0.002), and more likely to achieve target doses of beta-blocker (p<0.001) and target angiotensin-converting enzyme inhibitor/angiotensin receptor blockade/angiotensin receptor neprilysin inhibitor (p = 0.003).

**Table 2:** Baseline clinical characteristics in patients on short-term and long-term tele-HFC.

	All Patients (n = 201)	Short-Term (<6 months) Tele-Clinic (n = 87)	Long-Term (≥6 months) Tele-Clinic (n = 114)	p Value
Age (years)	56 ± 16	56 ± 15	55 ± 16	0.561
Male, no. (%)	135 (67%)	56 (64%)	79 (69%)	0.461
Left ventricular ejection fraction (%)	37 ± 18	35 ± 18	39 ± 17	0.052
Ischemic, no. (%)	73 (36%)	32 (37%)	41 (36%)	0.913
NYHA III-IV, no. (%)	40 (20%)	23 (26%)	17 (15%)	0.040†
Stage-D HF	45 (22%)	29 (33%)	16 (14%)	0.001†
HF with reduced EF, no. (%)	133 (66%)	66 (76%)	67 (59%)	0.010†
HF with improved EF, no. (%)	22 (11%)	4 (5%)	18 (16%)	0.002†
CIED, no. (%)	65 (42%)	32 (37%)	33 (29%)	0.240
Atrial fibrillation, no. (%)	57 (28%)	30 (35%)	27 (23%)	0.070
Medical treatment				
ACEI/ARB/ARNI, no. (%)	138 (89%)	60 (86%)	78 (92%)	0.230
50% of target dose	96 (62%)	35 (50%)	61 (72%)	0.006†
100% of target dose	48 (31%)	18 (26%)	30 (35%)	0.199
Beta-blockers, no. (%)	145 (94%)	64 (91%)	81 (95%)	0.329
50% of target dose	97 (62%)	34 (49%)	63 (74%)	0.001†
100% of target dose	65 (42%)	22 (32%)	43 (51%)	0.001†
MRA, no. (%)	95 (61%)	40 (57%)	55 (65%)	0.336
50% of target dose	95 (61%)	40 (57%)	55 (65%)	0.336
100% of target dose	59 (38%)	25 (36%)	34 (40%)	0.585
Furosemide, no. (%)	151 (75%)	75 (86%)	76 (67%)	0.002†
Furosemide >160 mg/day	27 (18%)	17 (23%)	10 (13%)	0.117
Thiazide/tolvaptan	14 (7%)	13 (15%)	1 (1%)	0.002†
BUN (mg/dL)	23 ± 12	23 ±11	23 ± 13	0.931
Creatinine (mg/dL)	1.45 ± 1.57	1.35 ± 2.45	1.53 ± 1.67	0.551
Duration of tele-visits (days)	180 (4–195)	136 (4–160)	183 (180–195)	<0.001†

ACE-I indicates angiotensin-converting enzyme inhibitor; ARB, angiotensin receptor blockade; ARNI, angiotensin receptor neprilysin inhibitor; BNP, B-type natriuretic peptide; BUN, blood urea nitrogen; CIED, cardiac implantable electronic device; EF, ejection fraction; HF, heart failure; MRA, mineralocorticoid receptor antagonist; and NYHA, New York Heart Association functional class.

^†^Statistically significant.

## Discussion

We report a marked decrease in HF readmission rate during the peak of the COVID-19 pandemic regarding the ambulatory care of patients with chronic HF. This phenomenon was similar to that observed in cross-sectional studies of acute HF [3, 4]. These rapid reductions may be caused by patients’ fears of contracting COVID-19 when presenting to the hospital or lower exposure to influenza-associated with worsening HF during the social distancing policy. Furthermore, the COVID-19 pandemic may have urged patients to be more aware and enthusiastic about adherence to self-care. The Tele-HFC program allows management of congestion by changing diuretic or aquaretic dosages and continuous medical up-titration of neurohormonal blockades. We found that medical titration during the peak of the pandemic was not different from that before and after the pandemic. Notably, this marked decline in HF readmission was not observed in a similar period in 2019, suggesting that seasonal trends in HF admission were less likely to conform to this pattern.

Compared with previous reports on acute HF hospitalization [3, 4], the hallmarks of our cohorts are the availability of the subsequent HF outcomes after the pandemic lockdown and the homogeneity of the study population who previously had been managed in the HF clinic before the HF readmission endpoint. By contrast, the study patients with HF in previous cross-sectional observations or secular trends in acute HF hospitalization may have different backgrounds with regard to previous HF management or disease severity, and there have been no cohort data on follow-up outcomes in those patients who were admitted to hospital during the study periods [3, 4]. The observations on acute HF hospitalization in the previous reports [3, 4] mostly ended in April 2020; in our study, we observed the clinical outcomes until October 1, 2020. We found that HF readmissions and CV deaths did not significantly increase or surge after the pandemic lockdown over the median time of 163 days. These findings could be due to the effect of the Tele-HFC, where continuous monitoring and treatment changes carried on. Additionally, a significant increase in heart transplant numbers after the peak of the pandemic may have rescued the sicker patients in the cohort, alleviating HF readmission and mortality rates.

Interestingly, about 40% of patients in our study preferred to be managed by the Tele-HFC after the COVID-19 crisis. These patients were more likely to be stable HF patients, with higher LVEF, NYHA class I–II, and stage-C HF, requiring a lower dose of diuretics, and on optimal medical therapy. Previous studies showed that telemedicine might improve medical titration [7–19]. Our data support the notion that medical optimization, including decongestion management, continued even in the absence of a face-to-face clinic visit.

Our findings highlight a need for potential restructuring of ambulatory care of HF. It is imperative to identify patients at low risk or with more stable HF who are appropriate for a long-term Tele-HFC program. Our data imply that virtual or tele-clinic visits during the pandemic unmasked the unnecessary and unwise HF care models, particularly in patients with a stable clinical HF status. The integration of or conversion to digital technology or the tele-clinic should be considered for such patients, resulting in valuable resource allocation and cost reduction in HF care.

Our study has several limitations. First, our Tele-HFC program is run in a tertiary care academic center where the HF and transplant cardiologists, HF nurses, and pharmacists have extensive experience in managing patients, which could limit the applicability and generalizability of our findings. Second, the data analysis was stratified to patients with HF who had telehealth appointments during the specified periods and therefore may not be generalizable to other HF groups. Third, because ultimate patient management was based on the physician’s decision, the variation of management across the patient population may inevitably have occurred. Last, this study is limited by its retrospective nature.

## Conclusions

Hospitalization of ambulatory HF patients rapidly declined by approximately 70% during the peak of the COVID-19 pandemic. However, our findings demonstrated no rapid surge of HF readmission or CV deaths at 5 months after the pandemic lockdown in the setting of the Tele-HFC program and timely advanced HF therapy. Further studies on longer-term adverse outcomes in a larger study population with HF are necessary.

## Supporting information

S1 File This is the minimal data set file for Figs 1 and 2. (DOCX)
