# Supplementary material for: The impact of the coronavirus disease and Tele-Heart Failure Clinic on cardiovascular mortality and heart failure hospitalization in ambulatory patients with heart failure
Source: PLoS One. 2021 Mar 23;16(3):e0249043. doi: 10.1371/journal.pone.0249043 (PMC7987182; doi:10.1371/journal.pone.0249043)
Supplement: S1 File — (DOCX) [file pone.0249043.s001.docx]

**Figure 1**

|  | **2020** | | | | | |
| --- | --- | --- | --- | --- | --- | --- |
|  | Pre-COVID-19 | | Peak COVID-19 | | Post COVID | |
|  | n | % | n | % | n | % |
| total n | **208** |  | **201** |  | **200** |  |
| CV death | 3 | **1.44** | 1 | **0.4975** | 1 | **0.5** |
| Transplant | 4 | **1.92** | 0 | **0** | 9 | **4.5** |
| HF admission | 18 | **8.65** | 5 | **2.4876** | 7 | **3.5** |

|  | **2019** | | | | | | |
| --- | --- | --- | --- | --- | --- | --- | --- |
|  | Pre-COVID-19 equivalent | | Peak COVID-19 equivalent | | Post COVID equivalent | |  |
|  | n | % | n | % | n | % |  |
| total n | **276** |  | **269** |  | **260** |  |  |
| CV death | 4 | **1.45** | 5 | **1.86** | 4 | **1.54** |  |
| Transplant | 3 | **1.09** | 4 | **1.49** | 5 | **1.92** |  |
| HF admission | 24 | **8.70** | 23 | **8.55** | 24 | **9.23** |  |

**Figure 2**

|  | Patients with HF-rEF and HF-iEF | | | |  |  |  |  |
| --- | --- | --- | --- | --- | --- | --- | --- | --- |
|  | Year 2020 |  |  |  |  |  |  |  |
|  |  |  |  |  |  |  |  |  |
|  |  |  | **Beta-blocker (BB)** | | **≥ 50% target dose of BB** | | **100% target dose of BB** | |
|  | Patients with HF-rEF and HF-iEF |  | n | % | n | % | n | % |
| Pre COVID | 160 |  | 144 | **90** | 99 | **61.88** | 54 | **33.75** |
| Peak COVID | 155 |  | 145 | **93.5** | 97 | **62.58** | 65 | **41.96** |
| Post COVID | 154 |  | 146 | **94.81** | 109 | **70.78** | 69 | **44.81** |
|  |  |  |  |  |  |  |  |  |
| **Figure 2 (continued)** |  |  | **ACE-I/ARB/ARNI** | | **≥ 50% target dose of ACE-I/ARB/ARNI** | | **100% target dose of ACE-I/ARB/ARNI** | |
|  | Patients with HF-rEF and HF-iEF |  | n | % | n | % | n | % |
| Pre COVID | 160 |  | 132 | **82.5** | 100 | **62.5** | 49 | **30.63** |
| Peak COVID | 155 |  | 138 | **89.03** | 96 | **61.9** | 47 | **30.32** |
| Post COVID | 154 |  | 138 | **89.61** | 99 | **64.3** | 55 | **35.71** |
|  |  |  |  |  |  |  |  |  |
|  |  |  | **MRA** | | **≥ 50% target dose of MRA** | | **100% target dose of MRA** | |
|  | Patients with HF-rEF and HF-iEF |  | n | % | n | % | n | % |
| Pre COVID | 160 |  | 97 | **61.19** | 96 | **60** | 59 | **36.9** |
| Peak COVID | 155 |  | 95 | **61.29** | 95 | **61.29** | 59 | **38.1** |
| Post COVID | 154 |  | 95 | **61.69** | 95 | **61.69** | 60 | **38.9** |
|  |  |  |  |  |  |  |  |  |
